# Ascl1 phospho-status regulates neuronal differentiation in a *Xenopus* developmental model of neuroblastoma

**DOI:** 10.1242/dmm.018630

**Published:** 2015-05-01

**Authors:** Luke A. Wylie, Laura J. A. Hardwick, Tatiana D. Papkovskaia, Carol J. Thiele, Anna Philpott

**Affiliations:** ^1^ Department of Oncology, Hutchison/MRC Research Centre, University of Cambridge, Cambridge Biomedical Campus, Cambridge CB2 0XZ, UK; ^2^ Pediatric Oncology Branch, Center for Cancer Research, NCI, CRC-1W-3940, 10 Center Dr. MSC-1105, Bethesda, MD 20892, USA

**Keywords:** Ascl1, *Xenopus*, Cell cycle, Development, Differentiation, Neuroblastoma

## Abstract

Neuroblastoma (NB), although rare, accounts for 15% of all paediatric cancer mortality. Unusual among cancers, NBs lack a consistent set of gene mutations and, excluding large-scale chromosomal rearrangements, the genome seems to be largely intact. Indeed, many interesting features of NB suggest that it has little in common with adult solid tumours but instead has characteristics of a developmental disorder. NB arises overwhelmingly in infants under 2 years of age during a specific window of development and, histologically, NB bears striking similarity to undifferentiated neuroblasts of the sympathetic nervous system, its likely cells of origin. Hence, NB could be considered a disease of development arising when neuroblasts of the sympathetic nervous system fail to undergo proper differentiation, but instead are maintained precociously as progenitors with the potential for acquiring further mutations eventually resulting in tumour formation. To explore this possibility, we require a robust and flexible developmental model to investigate the differentiation of NB's presumptive cell of origin. Here, we use *Xenopus* frog embryos to characterise the differentiation of anteroventral noradrenergic (AVNA) cells, cells derived from the neural crest. We find that these cells share many characteristics with their mammalian developmental counterparts, and also with NB cells. We find that the transcriptional regulator Ascl1 is expressed transiently in normal AVNA cell differentiation but its expression is aberrantly maintained in NB cells, where it is largely phosphorylated on multiple sites. We show that Ascl1's ability to induce differentiation of AVNA cells is inhibited by its multi-site phosphorylation at serine-proline motifs, whereas overexpression of cyclin-dependent kinases (CDKs) and MYCN inhibit wild-type Ascl1-driven AVNA differentiation, but not differentiation driven by a phospho-mutant form of Ascl1. This suggests that the maintenance of ASCL1 in its multiply phosphorylated state might prevent terminal differentiation in NB, which could offer new approaches for differentiation therapy in NB.

## Abstract (precis)

**Highlighted Article:** Neuroblastoma cells are stalled at a developmental stage at which they express high ASCL1. Multi-site phosphorylation of ASCL1, driven by elevated N-Myc and CDK activity, limits noradrenergic precursor and NB cell differentiation.

## INTRODUCTION

Neuroblastoma (NB) is a tumour of the autonomic nervous system and is the most common cancer diagnosed in the first year of life. Although the incidence of NB is considered to be relatively low, with 10.2 cases per million children under the age of 15, it accounts for 15% of all paediatric cancer mortality (Ries et al., 1999). A stark dichotomy is seen among NB cases, with some forms undergoing spontaneous regression and showing a strong response to treatment whereas others progress to fatal metastasis and fail modern therapy (Maris, 2010). Although substantial strides have been made in the last 20 years, current treatment regimens are still inadequate or unsuccessful for 40% of patients (Matthay et al., 1999). Early genetic studies into NB revealed some large-scale chromosomal rearrangements and amplifications in some tumours; most notably *MYCN* amplification was found to be consistently associated with poor prognosis in NB (Schwab et al., 1983). However, although large-scale rearrangements and chromosomal losses are sometimes observed, NB seems to lack a consistent set of mutations as seen in many other cancers. Indeed, more recent large-scale sequencing studies have revealed that NB tumours have an average of only 12 amino-acid-changing mutations per tumour, with the highest recurring mutated gene, *ALK*, being mutated in only 6-10% of tumours, suggesting that the genome of NB is largely intact (Molenaar et al., 2012; Pugh et al., 2013).

If NB formation is not initiated by consistent genetic mutations, then one possibility is that NB results from cells that are developmentally arrested during noradrenergic (NA) neuron differentiation, when the epigenetic landscape of precursor cells promotes proliferation and inhibits terminal differentiation. Such a scenario could explain why NB is seen largely only during infancy, because the initial deviation from normal development toward tumorigenesis can only occur during a specific time during development. NB is thought to be derived from precursor cells of the sympathetic nervous system (sympathetic progenitors) based on the locations of primary tumours (Maris et al., 2007) and the similar expression profile of NB to sympathetic progenitors (De Preter et al., 2006). One might hypothesise that NA precursor cells bypass their normal progression to terminal differentiation but instead maintain their proliferative potential for an extended period of time. As these cells continue to proliferate they might acquire further epigenetic or genetic changes that result in cancer. Supporting evidence for this view is the therapeutic use of retinoic acid, a known inducer of neuronal differentiation *in vivo*, which can result in neuronal differentiation and cell cycle arrest of NB cells *in vitro* (Matsuo and Thiele, 1998; Thiele et al., 1985) and improved survival in patients undergoing intensive chemotherapy and radiation treatment (Matthay et al., 1999). In addition, recent work has demonstrated that genes associated with differentiation and inhibition of proliferation are epigenetically suppressed in NB via PRC2, suggesting that there might be an epigenetic block in these pathways rather than a genetic one (Wang et al., 2012). However, existing therapies for NB are only partially effective in aggressive disease and result in significant long-term complications for NB patients, so more targeted therapies are required.

**TRANSLATIONAL IMPACT**

**Clinical issue**

Neuroblastoma (NB) is a paediatric cancer of infancy that occurs in roughly 10.2 children per million. Although rare, NB still accounts for 15% of all paediatric cancer mortality owing to its clinically unfavourable outcome. Treatment for patients with poor prognosis, often associated with *MYCN* (v-myc avian myelocytomatosis viral oncogene neuroblastoma derived homolog) amplification, is inadequate in 40% of cases. Therefore, new avenues for therapy should be explored. Whereas most cancers arise from a rapid acquisition of gene mutations, the genome of NB is largely intact, suggesting a non-genetic cause for this disease. Given that NB only arises during infancy and shows many histological similarities with progenitor cells of the sympathetic nervous system, it could represent a cancer driven by a failure in the normal developmental programme. Understanding normal mechanisms that are involved in sympathetic noradrenergic neuron development could give insight into NB pathogenesis and might suggest new approaches to therapy.

**Results**

The authors describe a population of anteroventral noradrenergic (AVNA) cells within *Xenopus* embryos that share common characteristics with cells of the sympathetic nervous system of mammals, the likely cell of origin for NB. They show that AVNA cells derive from the neural crest and express a series of noradrenergic genetic markers that are shared with NB cells. Interestingly, they show that one of these genes, *Ascl1* – achaete-scute complex-like 1, which encodes a transcriptional driver of neurogenesis – is transiently expressed during AVNA cell differentiation, but is aberrantly maintained in NB. Building on previous studies characterising post-translational regulation of Ascl1, the authors demonstrate that, although Ascl1 is present in NB, it is phosphorylated on multiple serine-proline sites and this inhibits its ability to drive AVNA cell differentiation. In addition, the authors use the AVNA cell model system to test how increased cyclin-dependent kinase (CDK; involved in cell cycle regulation) and MYCN activity, found in NB, affects Ascl1's ability to induce differentiation. They find that ectopic AVNA cell differentiation driven by Ascl1 is inhibited by increased CDK and MYCN activity, and this inhibition depends on the phosphorylation of Ascl1.

**Implications and future directions**

This work has two important implications. First, it shows that the AVNA model system offers a uniquely flexible system for analysing the initial stages of sympathetic nervous system development, during which NB is thought to arise. Secondly, it suggests that maintenance of phosphorylation of ASCL1 on serine-proline sites in NB might contribute to a failure of these cells to continue down their normal developmental pathway and undergo terminal differentiation. Inhibition of ASCL1 phosphorylation could therefore represent a new strategy to enhance cell differentiation as a potential therapy in NB.

If NB is a tumour partially derived from a failure to undergo the appropriate developmental programme of differentiation, it becomes imperative to understand what that developmental programme is, and where it becomes arrested or disrupted in NB. The sympathetic nervous system is derived from the neural crest (Howard, 2005; Rohrer, 2011). These cells are difficult to characterise in mammals because of their developmentally controlled migration and their inaccessibility. To better understand the causes of NB and as a platform to explore novel therapeutic approaches, research would benefit greatly from a tractable experimental model to study normal NA neuron differentiation and how it can be perturbed by manipulations mimicking the situation in NB.

A number of developmental and embryonic stem (ES) cell model systems are available to study normal development of the NA nervous system, and these have been used to develop genetic models of NB (Berry et al., 2012; Weiss et al., 1997; Zhu et al., 2012). However, these models have not been widely used to understand when and where during development NB cells arrest, and which non-genetic intra- and extracellular changes in the developmental environment might contribute to perturbation of the NA differentiation programme. For such studies, one must combine experimental embryology with the ability to perturb levels of cell cycling and gene expression to investigate the effect on NA neuron differentiation programmes. Therefore, a model system that is well-characterised, allows for alteration in gene expression and protein biochemistry, and permits analysis of large numbers of embryos at multiple developmental stages is required. These requirements are best represented by investigation of NA neuron development in tadpoles of the frog *Xenopus laevis*.

Fortuitously, recent work has identified a set of NA precursor cells in *Xenopus* embryos that express similar markers to the developing mammalian sympathetic nervous system from which NBs arise (Parlier et al., 2008). These cells express Ascl1, Phox2a, Hand2 and tyrosine hydroxylase (TH), and are dependent on BMP signalling, mirroring development of the sympathetic nervous system in mammals (Howard, 2005; Rohrer, 2011). Here, we demonstrate that this population of *Xenopus* NA cells is derived from migrating neural crest, as is their mammalian counterpart. We further explore the utility of this *Xenopus* system for characterising normal NA development and differentiation of NA cells within the signalling context of NB, by experimental manipulation of cyclin-dependent kinase (CDK) and N-Myc levels on NA cell differentiation, mimicking similar perturbations found in NB.

## RESULTS

### Migration of neural crest is required for expression of NA markers in the anteroventral region

**Fig. 1.**
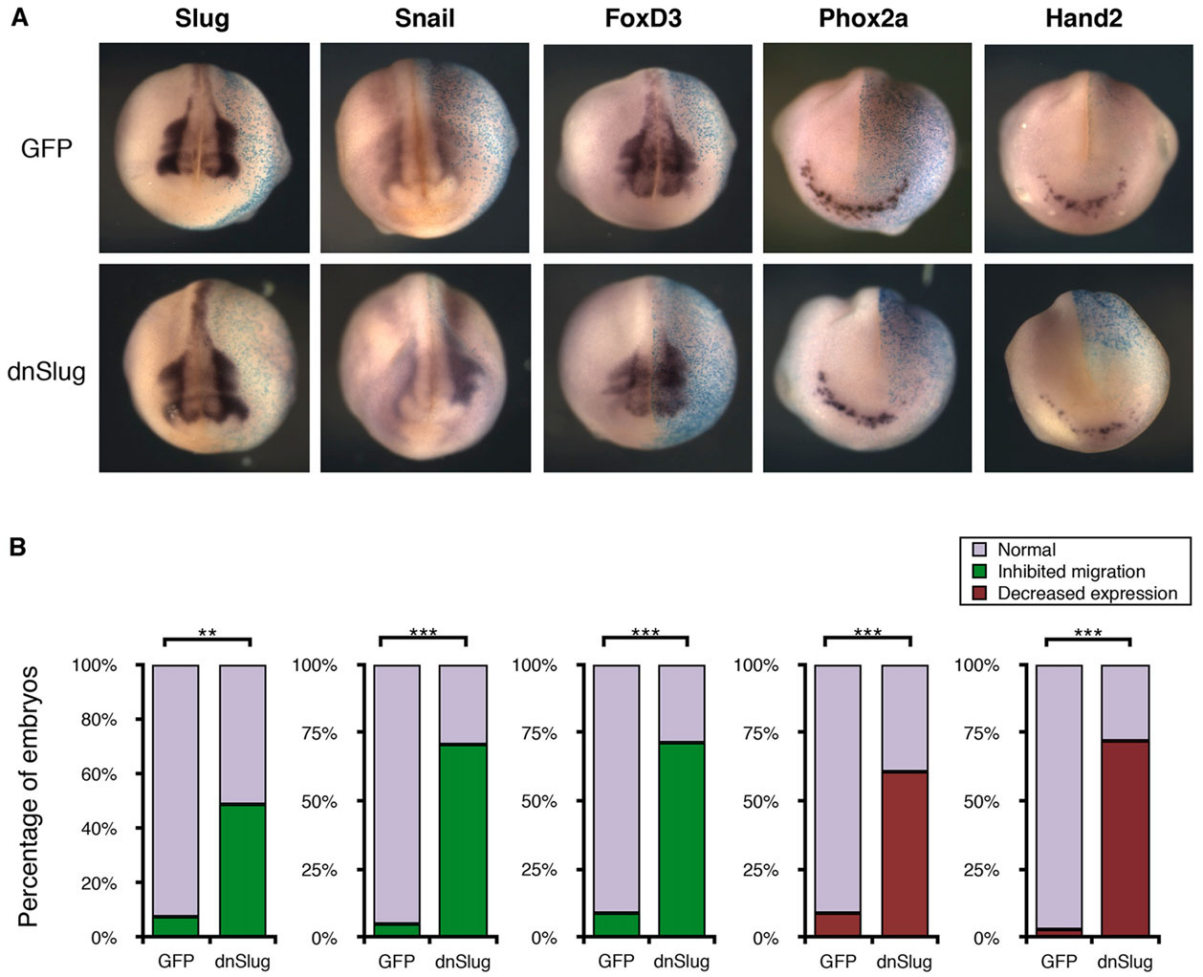
**Migration of neural crest is required for expression of noradrenergic markers in the anteroventral region.** *Xenopus* embryos were injected in one cell at the two-cell stage with β-galactosidase (light blue; A) as a tracer along with either GFP as a control, or mRNA encoding a dominant-negative form of Slug (dnSlug) to inhibit neural crest migration; cells derived from the injected cell are shown in the side to the right in A. (A) Embryos were fixed at stage 17/18 and subject to *in situ* hybridisation for the neural crest markers Slug, Snail and FoxD3, and noradrenergic (NA) markers Phox2a and Hand2. (B) Embryos were scored compared to the uninjected side based on inhibition of migration for neural crest markers and reduced expression of NA markers (see supplementary material Fig. S3). For scoring data, *n*=28-45. Kruskal–Wallis non-parametric ANOVA was performed, comparing GFP control with dnSlug-injected embryos (**P<0.0005 and ***P<0.000005).

Parlier and colleagues identified a population of cells within the anteroventral region of the *Xenopus* embryo that express a series of markers [Ascl1 (Xash1), Phox2a, Hand2 and TH], suggesting that they are an early component of the sympathetic nervous system, and are hereby referred to as anteroventral noradrenergic (AVNA) cells (Parlier et al., 2008). However, it was not clear whether these cells arise *de novo* after induction by the underlying heart field or whether they are, in fact, derived from migrating neural crest, as the NB cells of origin are known to be (Howard, 2005; Rohrer, 2011; Sarkar and Howard, 2006). To test this, we simultaneously injected a dominant-negative form of Slug (dnSlug), which inhibits neural crest migration (LaBonne and Bronner-Fraser, 2000), along with β-galactosidase, serving as a tracer for injection, in one cell of the two-cell-stage embryo, and then stained for the AVNA markers Phox2a and Hand2 by *in situ* hybridisation. When comparing the dnSlug-injected to the uninjected side of the embryo, neural crest markers Slug, Snail and FoxD3 displayed more intense staining and remained near the dorsal edge of the neural folds, demonstrating inhibition of neural crest migration (Fig. 1A,B). In the presence of dnSlug, we observed reduced expression of NA markers Phox2a and Hand2, which are normally found in the ventral region (Fig. 1A,B). Our data therefore indicate that the AVNA cells in *Xenopus* are derived from neural crest and might represent a population of cells analogous to the cells from which NB is thought to derive.

### Ascl1 is expressed transiently during NA cell development in the anteroventral region

**Fig. 2.**
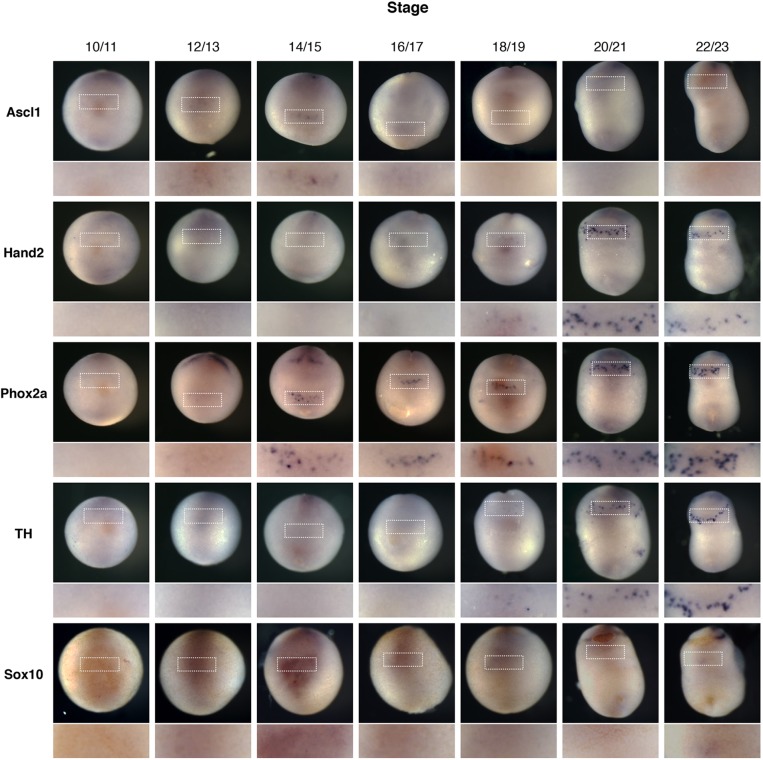
**Ascl1 is expressed transiently during noradrenergic development in the anteroventral region.** *Xenopus* embryos were fertilised and allowed to develop to the developmental stage indicated before fixing and staining by *in situ* hybridisation for noradrenergic (NA) neuron markers Ascl1, Hand2, Phox2a and TH, along with the neural crest marker Sox10. Embryos are all orientated with the ventral side imaged and head to the top. The anteroventral region where AVNA cell markers are expressed is expanded in the lower panel.

If NB cells do represent a stalled stage of NA cell differentiation, it is not clear at which developmental stage the cells are arrested. To provide a framework to investigate this, we built on the initial analysis of Parlier and colleagues (2008) to further characterise the developmental expression of the AVNA markers Phox2a, Hand2 and Ascl1 in *Xenopus* embryos by *in situ* hybridisation (Fig. 2). Phox2b, although important during development of the sympathetic nervous system in mammals (Pattyn et al., 1999; Unsicker et al., 2005), was not expressed within the anteroventral region of *Xenopus* embryos (Talikka et al., 2004). In addition, embryos were also stained with Sox10, a marker of neural crest and early peripheral nervous system development (Britsch et al., 2001; Southard-Smith et al., 1998).

Firstly, Ascl1, Hand2, Phox2a and TH were all expressed within the same anteroventral region of the embryo, consistent with the identification of these cells as NA neurons. Furthermore, some Sox10 expression was observed within the anteroventral region at stage 14/15, which is consistent with its previously described role in development of the peripheral nervous system and induction of Ascl1 (Kim et al., 2003; Sonnenberg-Riethmacher et al., 2001), although this expression was notably less pronounced than staining in the central nervous system (supplementary material Fig. S1) (Honoré et al., 2003). Phox2a and Hand2 were first expressed at stage 12/13 and 16/17, respectively, and expression remained at least until stage 23. Ascl1 was, however, expressed transiently, first appearing at stage 14/15 and being downregulated by stage 16/17, suggesting that its role during development is transient, much like its mammalian counterpart (Guillemot et al., 1993). Thus, Ascl1 is expressed at the onset of NA neuron differentiation but is rapidly downregulated, and hence is well placed to play a role in the progenitor maintenance versus differentiation decision specifically at these early developmental stages.

### p27Xic1 knockdown by morpholino injection inhibits neurogenesis

**Fig. 3.**
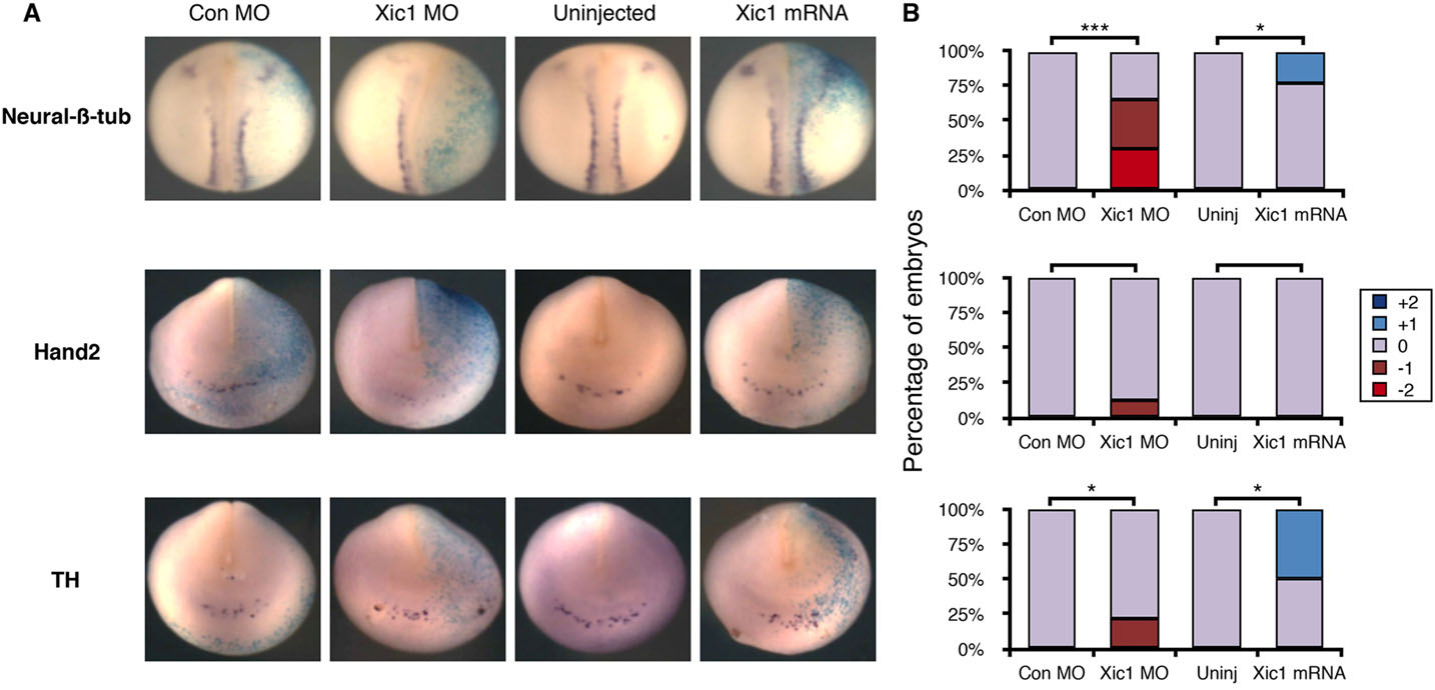
**p27Xic knockdown by morpholino injection inhibits neurogenesis.** *Xenopus laevis* embryos were injected in one cell at the two-cell stage with β-galactosidase (light blue) as a tracer along with either *Xic1* mRNA or *Xic1* antisense morpholino (MO) as indicated; cells derived from the injected cell are shown in the side to the right in A. (A) At stage 19/20, embryos were subject to *in situ* hybridisation to detect neural-β-tubulin, Hand2 and TH. Con MO, control MO. (B) Embryos were scored for marker expression on the injected side relative to the uninjected side (see supplementary material Fig. S3 for scoring scheme). For scoring data, *n*=12-34. Kruskal–Wallis non-parametric ANOVA was performed on control MO compared to *Xic1* MO and uninjected compared to *Xic1* mRNA (*P<0.05 and ***P<0.000005).

Primary neurons are the first neurons to differentiate in the developing *Xenopus* embryo and form in three stripes either side of the mid-line within the dorsal neural plate (Vernon et al., 2003). Expression of the CDK inhibitor (CDKI) p27Xic1 is absolutely required for primary neuron differentiation, as assayed by staining for neural-β-tubulin (Vernon et al., 2003). Conversely, when p27Xic1 is overexpressed, extra primary neurons form at the expense of maintenance of neural precursors (Vernon et al., 2003). Similar to primary neurons, almost all mammalian neurons become post-mitotic when they differentiate (Hardwick and Philpott, 2014). However, sympathetic neurons are unique in that they retain their ability to proliferate even after they express markers of mature differentiation (Ernsberger et al., 1989). We investigated whether the formation of AVNA cells has a lesser requirement for cell cycle exit compared with primary neurons, and hence has this in common with developing mammalian NA neurons. To test this, we inhibited p27Xic1 translation using an antisense morpholino (MO), which has been previously validated by western blotting and rescue experiments in *Xenopus* embryos (Vernon et al., 2003), or we increased the level of p27Xic1 by mRNA injection. We then assayed for expression of neural-β-tubulin, a marker of primary neuron differentiation, and also the later markers of NA differentiation Hand2 and TH (Fig. 3). As we have previously seen, *p27Xic1*-MO injection led to reduced expression of neural-β-tubulin on the injected side, demonstrating a loss of primary neurons compared with the contralateral uninjected side (Vernon et al., 2003). This occurred in at least 25% of the injected embryos, with an additional almost 30% of embryos showing an observable decrease in neural-β-tubulin expression. The decrease in neural-β-tubulin is consistent with a requirement for CDKIs in primary neuron differentiation. However, a modest decrease in Hand2 and TH induced by the *p27Xic1* MO was detected in only 10-25% of embryos. This indicates that primary neurons have a greater requirement for CDKIs to support differentiation than do AVNA cells.

We then investigated whether raising the level of p27Xic1 could enhance AVNA cell specification and differentiation. High levels of *p27Xic* mRNA injected into one cell of two-cell embryos resulted in rapid cell cycle exit and cell death (Vernon et al., 2003). However, lower levels of *p27Xic1* slowed but did not arrest the cell cycle and resulted in extra primary neurons differentiating within the neural plate, particularly obvious at open neural plate stages (Vernon et al., 2003) but also visible after neural tube closure in a minority of embryos (Fig. 3). Injection of a low level of *p27Xic1* mRNA had no effect on expression of Hand2, but there was a modest increase in TH expression in *p27Xic1*-injected embryos in approximately 50% of the embryos (Fig. 3). Taken together, these results indicate that the differentiation of AVNA cells is less dependent on the presence of CDKIs than is primary neurogenesis. This is consistent with previous evidence showing that NA neurons can express differentiation markers while still maintaining their ability to proliferate (Ernsberger et al., 1989). Nevertheless, despite a reduced requirement for cell cycle exit and CDK inhibition for AVNA neurogenesis compared with primary neuron formation, elevated levels of CDKIs can still modestly promote AVNA cell differentiation. This led us to consider what influences the cell cycle might have on differentiation of both sympathetic NA cells during development and on NB cells under pathological conditions, as well as potential mechanisms for such an influence.

### NB expresses the proneural protein Ascl1 and NA markers

**Fig. 4.**
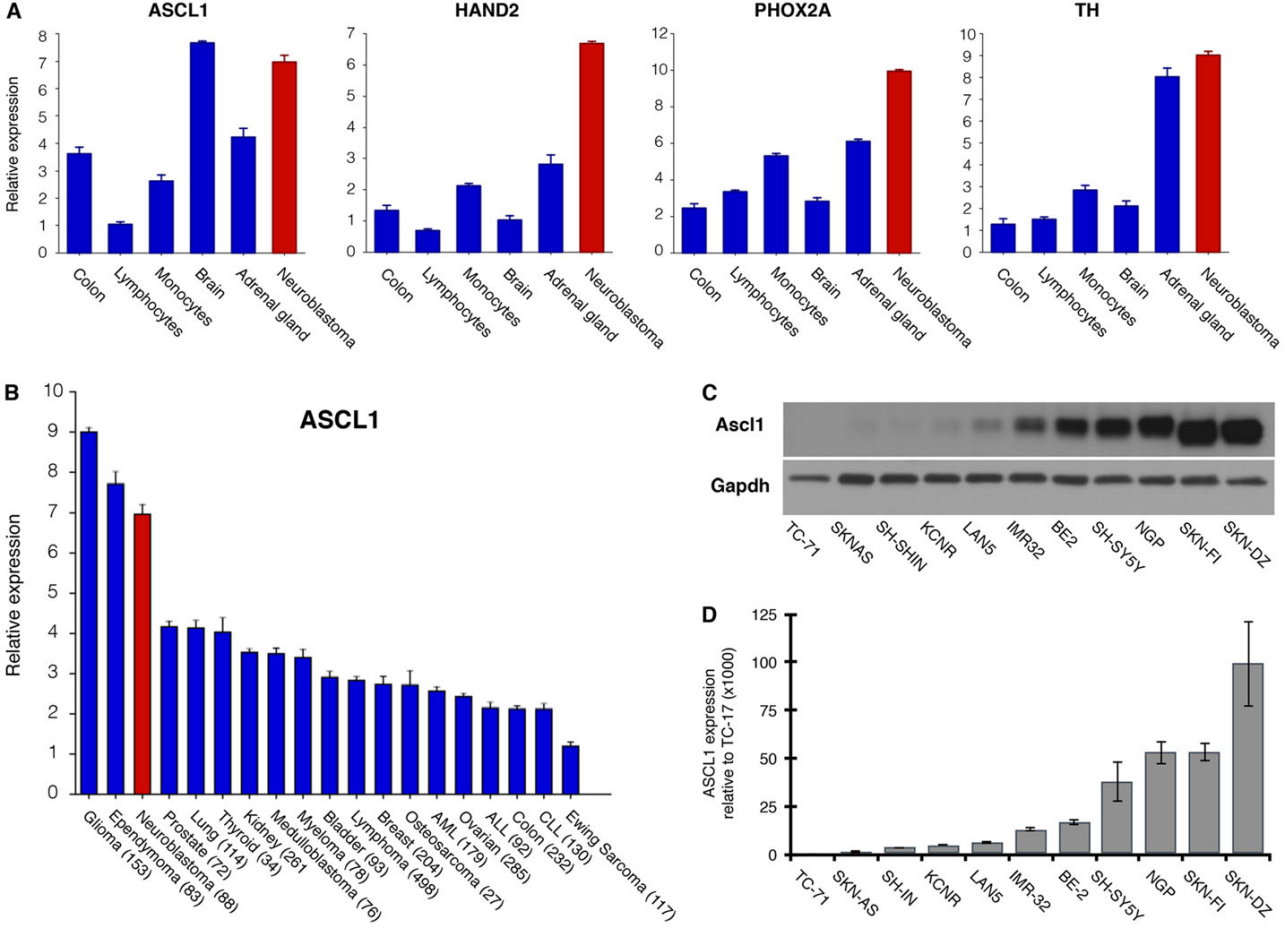
**Neuroblastoma expresses the proneural protein ASCL1 and noradrenergic markers.** Relative expression of ASCL1 and NA markers in neuroblastoma (NB) primary tumours and normal tissue (A), and of ASCL1 across tumour types (B), were determined by analysing publicly available microarray data (see Materials and Methods). The number of tumour samples used in the analysis are indicated next to the tumour type in B. ASCL1 expression in NB cell lines and the Ewing sarcoma line TC-71, as a negative control, was determined by western blot (C) and qPCR (D).

As described above, one possibility is that NB arises partially as a consequence of arrested differentiation of sympathetic NA cells in the developing peripheral nervous system. Determining when this arrest occurs within the differentiation programme would prove useful because it would help to identify potential pathways that might be involved in the initial steps of NB tumorigenesis. Our data indicate that *Xenopus* AVNA cells might represent an analogous population to the sympathetic lineage from which NB arises that would be more amenable to experimental manipulation than mammalian NA precursors. We compared gene expression in NB to ascertain whether this resembled an expression profile similar to a particular stage of normal NA neuron development. We undertook this by analysing the expression of NA markers that we characterised in *Xenopus* AVNA development (Ascl1, Hand2, Phox2a and TH; Fig. 2) in NB primary tumours, using publicly available microarray data (Fig. 4). We found that NB primary tumours have high expression of PHOX2A, HAND2 and TH. Interestingly, although only transiently expressed in AVNA development, NBs also have high levels of ASCL1 (Fig. 4A). This suggests that NB is either stalled at a stage in development after NA markers have been expressed, but prior to ASCL1 downregulation, or that ASCL1 is aberrantly actively maintained at a high level.

We then investigated ASCL1 expression in a number of human neural-derived tumours, again using publically available microarray data (Fig. 4B). As well as a high level of expression in NBs, ASCL1 levels were also elevated in other neural-based tumours (glioma and ependymoma), whereas other tumour types showed lower levels (Fig. 4B) (Rheinbay et al., 2013). Although ASCL1 seemed to be expressed across a number of NB primary tumours, we next verified this in a panel of NB cell lines both at the mRNA and protein level. All NB cells tested expressed variable levels of protein, with some cell lines having substantially higher protein levels than others (Fig. 4C). *ASCL1* mRNA was also variably expressed in NB cell lines (Fig. 4D), with the levels of mRNA correlating with the levels of protein in the different NB cell lines. High levels of ASCL1 are perhaps surprising because ASCL1 is most well-characterised for its role in driving neuronal differentiation (Pang et al., 2011; Vierbuchen et al., 2010), but NB cells continue to proliferate despite having readily detectable levels of ASCL1. However, ASCL1 has recently been shown to play a functional role in maintaining ‘stemness’ in murine cortical neural stem cells (Castro et al., 2011) and in human glioblastoma (Rheinbay et al., 2013), indicating that ASCL1 expression does not always result in neuronal differentiation. We have explored why NA neurons expressing high levels of ASCL1 might nevertheless be inhibited from undergoing neuronal differentiation, using NB cells and our *Xenopus* AVNA system.

The ability of Ascl1 to drive neuronal differentiation in the central nervous system is compromised by high levels of CDKs. We have previously shown that Ascl1 is phosphorylated on multiple serine-proline (SP) sites in *Xenopus* embryos and by CDKs *in vitro* (Ali et al., 2014). Phosphorylation on these sites limits its ability both to induce ectopic primary neurogenesis in *Xenopus* embryos and to promote maturation of neurons generated from human fibroblasts by a transcription-factor-mediated reprogramming cocktail (Vierbuchen et al., 2010). To test whether phospho-regulation of ASCL1 protein might be occurring in NB cells, we investigated the phospho-status of ASCL1 in multiple NB cell lines.

### ASCL1 is phosphorylated in NB lines

**Fig. 5.**
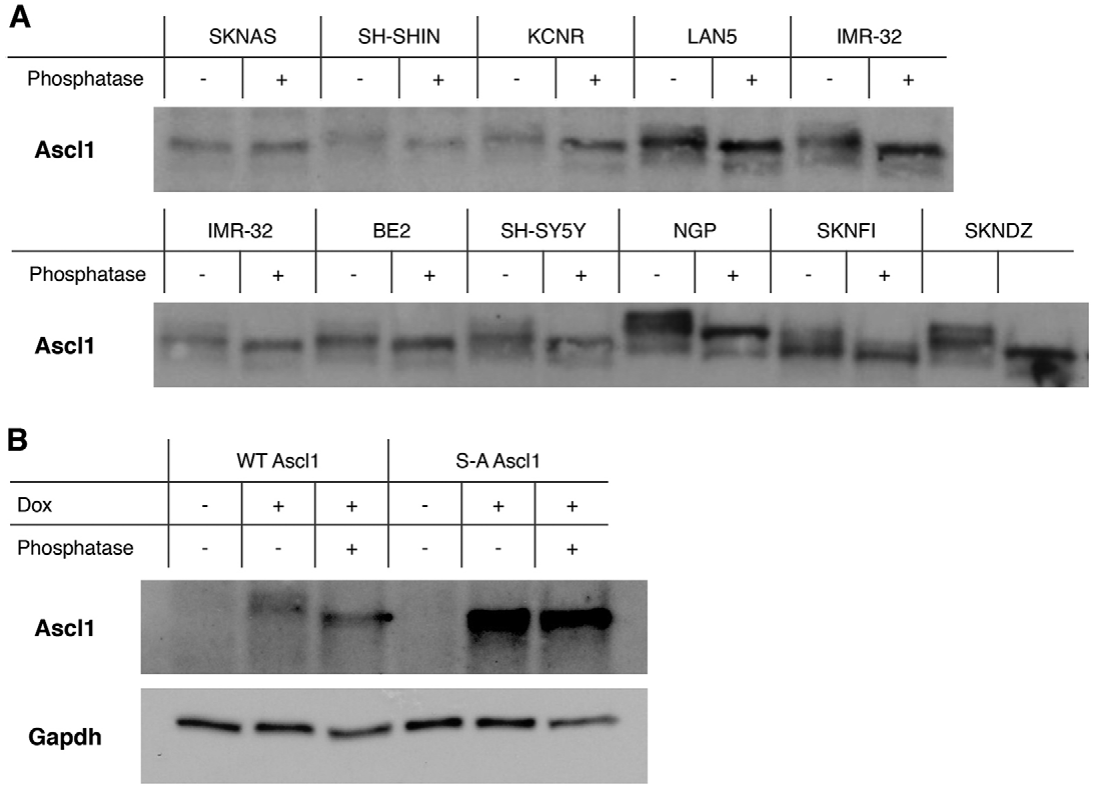
**ASCL1 is phosphorylated on multiple sites in** **neuroblastoma** **cells.** (A) Protein extracts from Fig. 4 were normalised based on ASCL1 expression and treated with and without lambda phosphatase. Samples were then separated by SDS PAGE and subjected to western blotting for ASCL1 protein. (B) Human ASCL1 or phospho-mutant S-A ASCL1 was overexpressed in SY5Y cells, and treated with and without lambda phosphatase. The slower migration observed on SDS PAGE can be attributed to phosphorylation on SP sites.

*ASCL1* mRNA and protein was detectable in all NB cell lines tested but was found to be expressed at differing levels (Fig. 4C,D). To investigate potential phosphorylation of ASCL1 in NB cells, we used an assay to detect retarded migration on SDS polyacrylamide gel electrophoresis (SDS PAGE), comparing results from before and after phosphatase treatment, because retarded migration has previously been shown to result from phosphorylation on SP sites (Ali et al., 2011). To facilitate this analysis, protein samples from Fig. 4C were loaded normalised to Ascl1 expression, allowing for clear ASCL1 protein detection in all lines. Migration of ASCL1 on SDS PAGE was then compared with and without treatment with the broad-spectrum lambda phosphatase to demonstrate ASCL1 phosphorylation status (Fig. 5A). In all NB cell lines tested, with the exception of SKNAS, ASCL1 migration was enhanced by treatment with phosphatase. In several cell lines, more than one retarded phospho-form was observed (e.g. NGP, SKNDZ, SH-SYHY). In addition, it is noteworthy that different cell lines showed a different pattern of migration of ASCL1 phospho-forms (e.g. compare NGP with SKNFI), indicating that the pattern of post-translational modification of ASCL1 might differ from cell line to cell line. We also observe that further post-translational modification of ASCL1 occurs in some lines that is not sensitive to phosphatase under the current conditions (e.g. in NGP cells).

To determine whether the observed change in migration on SDS PAGE was due to phosphorylation of serine-proline motifs in ASCL1, we created inducible SH-SY5Y cell lines that express either human WT ASCL1 or a phospho-mutant form of ASCL1 in which all five serine-proline motifs (see supplementary material Fig. S2), potential targets of CDK-mediated phosphorylation (Ali et al., 2014), are mutated to alanine-proline [serine to alanine (S-A) ASCL1]. Expression of ASCL1 was then induced for 24 h and cell extracts treated with and without phosphatase to observe any potential change in ASCL1 SDS PAGE migration (Fig. 5B). WT ASCL1 migrated more slowly than the phosphatase-treated protein, whereas S-A ASCL1 migrated at the same level as the de-phosphorylated WT ASCL1 protein and showed no detectable sensitivity to phosphatase. This is consistent with phosphorylation of ASCL1 on SP sites in NB cells. Phosphorylation on these sites has previously been shown to be mediated by the CDK2 kinase *in vitro* (Ali et al., 2014).

### CDK activity inhibits AVNA cell differentiation induced by WT ASCL1 but not S-A ASCL1

Although AVNA cells seem to be less dependent on cell cycle exit for differentiation compared to primary neurons, overexpression of the CDKI p27Xic1 nevertheless promotes enhanced TH expression (Fig. 3), indicating that CDK activity can influence AVNA cell differentiation. This might be by CDK-dependent phosphorylation and inactivation of ASCL1 (Ali et al., 2014). Elevated CDK levels are known to inhibit neuronal differentiation in the CNS of *Xenopus* and mammals (Lange et al., 2009; Richard-Parpaillon et al., 2004). Our previous work demonstrated that high CDK activity inhibits ASCL1's ability to drive primary neurogenesis by phosphorylation on SP sites of WT ASCL1, whereas S-A ASCL1 can induce primary neuron differentiation even when CDK kinase levels are raised (Ali et al., 2014). We hypothesized that a similar mechanism might regulate ASCL1's ability to induce AVNA cell differentiation. We first tested whether elevated CDK levels do indeed inhibit the differentiation of AVNA cells in *Xenopus*.

**Fig. 6.**
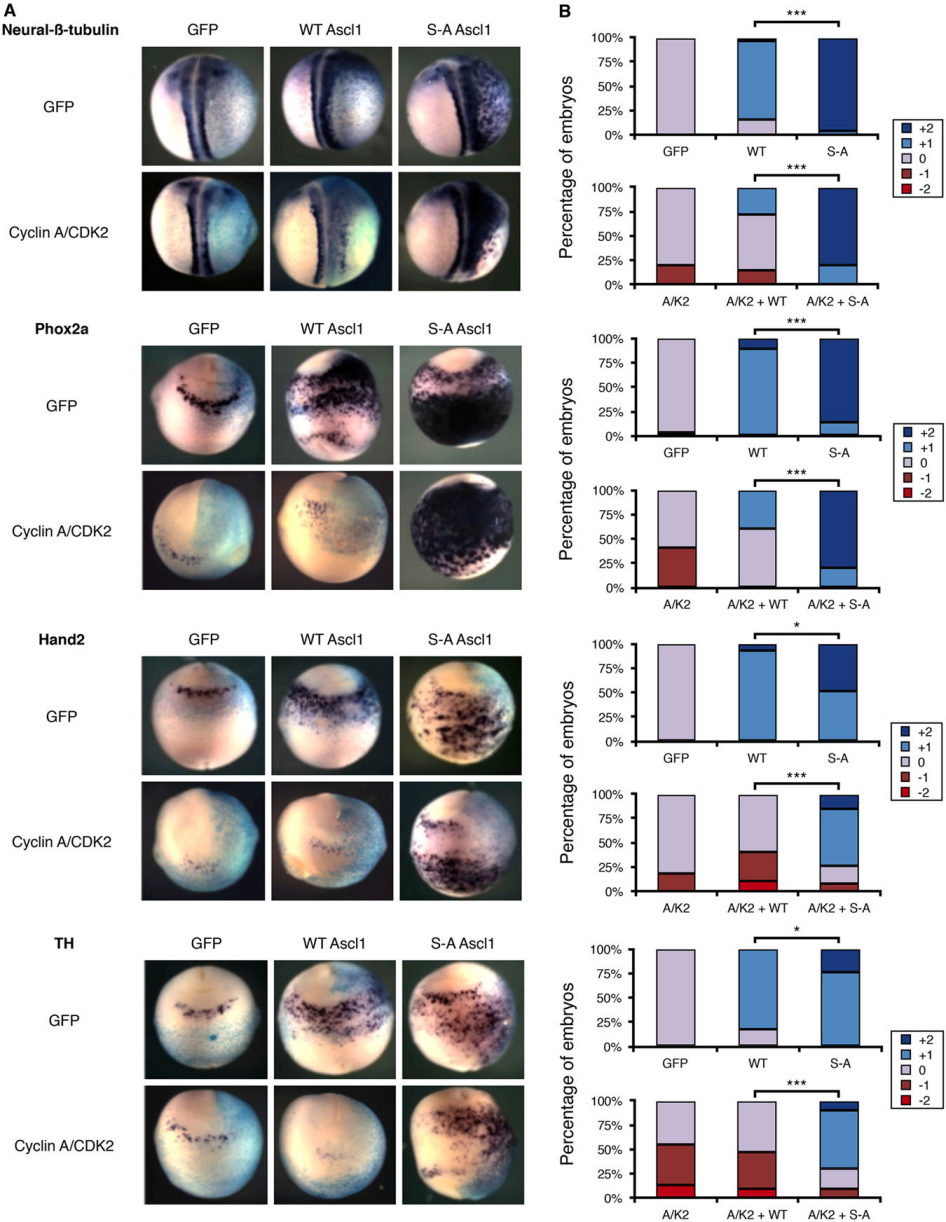
**CDK activity inhibits AVNA cell differentiation induced by WT ASCL1 but not S-A ASCL1.** *Xenopus* embryos were injected in one cell at the two-cell stage with β-galactosidase (light blue; A) as a tracer along with mRNA encoding cyclin A/CDK2 and/or mouse WT/S-A Ascl1, as indicated; cells derived from the injected cell are shown in the side to the right in A. (A) Embryos were subject to *in situ* hybridisation for AVNA markers at stage 18-19, as labelled. (B) Embryos were scored for marker expression comparing the injected and uninjected side (see supplementary material Fig. S3 for scoring scheme). For scoring data, *n*=20-32. Kruskal–Wallis non-parametric ANOVA was performed comparing WT ASCL1 with S-A ASCL1 without and with cyclin A/CDK2 (*P<0.05 and ***P<0.000005).

To enhance CDK levels in *Xenopus*, we co-injected mRNA encoding cyclin A2 and CDK2 into one cell of a two-cell embryo; this has been previously shown to enhance kinase activity and cell cycling (Ali et al., 2014; Richard-Parpaillon et al., 2004), and to directly phosphorylate Ascl1 (Ali et al., 2014). Cyclin and CDK overexpression delays but does not prevent primary neurogenesis (Richard-Parpaillon et al., 2004), and led to a modest but clearly observable decrease in endogenous Hand2 and Phox2a expression in some embryos (Fig. 6). We also observed a more marked reduction in TH expression, consistent with elevated CDK levels suppressing particularly later stages of AVNA cell differentiation (Fig. 6).

We then looked at the effect of elevated cyclin A2 and CDK2 levels on the differentiation of neurons generated by overexpression of Ascl1, which results in ectopic generation of two distinct cell populations. As previously reported, *Ascl1* mRNA injection resulted in increased expression of neural-β-tubulin dorsally in the embryo, corresponding to generation of extra primary neurons (Ali et al., 2014). Ectopic AVNA cells generated in the ventral region by Ascl1 overexpression do not express neural-β-tubulin, but do express Phox2a, Hand2 and TH, and so represent a population of additional AVNA cells (Parlier et al., 2008). This demonstrates that Ascl1 can drive differentiation of distinct populations of cells in different regions of the *Xenopus* embryo.

Cyclin A and CDK2 efficiently suppressed the generation of ectopic AVNA cells occurring after overexpression of WT Ascl1 (Fig. 6). However, S-A Ascl1 was largely insensitive to elevated CDK levels, still generating extensive ectopic Hand2-, Phox2a- and TH-expressing cells on the ventral side of the embryo. These results are consistent with elevated levels of CDKs inhibiting the formation of NA neurons by directly phosphorylating Ascl1 on SP sites and preventing its ability to drive differentiation (Ali et al., 2014).

### N-Myc inhibits AVNA cell differentiation induced by WT Ascl1 but not S-A Ascl1

Although aberrant high CDK activity is clearly associated with NB (Molenaar et al., 2009, 2008), one of the strongest markers of poor prognosis for individuals with NB is *MYCN* amplification (Brodeur, 2003). N-Myc expression driven by the TH promoter causes NB in mice (Weiss et al., 1997), suggesting that N-Myc is a critical effector of NB pathogenesis. Furthermore, even in non-*MYCN*-amplified tumours, a *MYCN*-amplified transcriptional signature is observed (Valentijn et al., 2012), and levels of c-Myc can also be elevated (Mestdagh et al., 2010), together suggesting that Myc signalling is an underlying driver of NB tumorigenesis. A role for c-Myc is established in early neural crest development in *Xenopus*, where it is an essential regulator of neural crest precursor cells (Bellmeyer et al., 2003). Moreover, N-Myc has been demonstrated to promote proliferation and inhibit differentiation in the CNS (Knoepfler et al., 2002), and seems to play a similar role in NB (Thiele and Israel, 1988; Thiele et al., 1985).

**Fig. 7.**
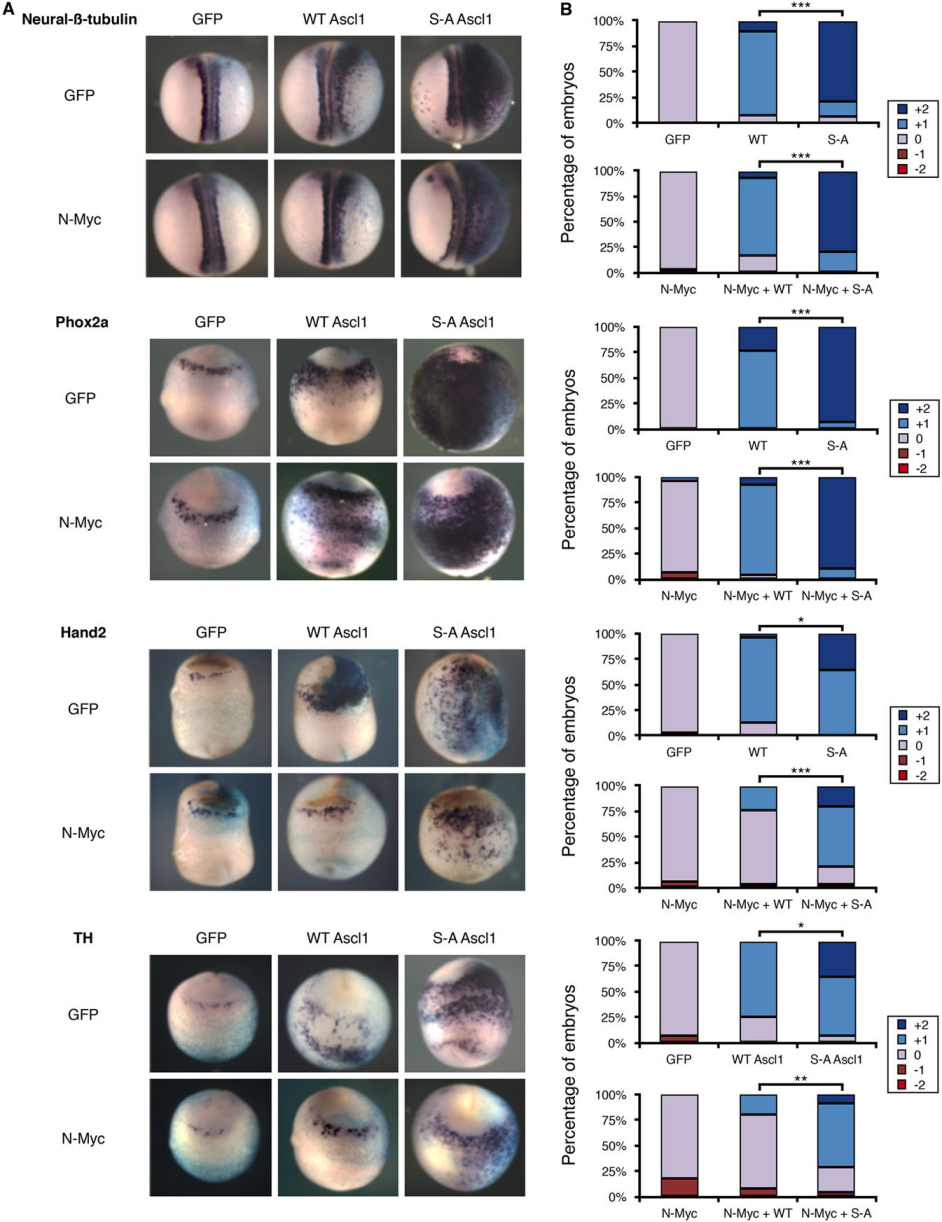
**N-Myc inhibits AVNA cell differentiation induced by WT ASCL1 but not S-A ASCL1.** *Xenopus* embryos were injected in one cell at the two-cell stage with β-galactosidase (light blue; A) as a tracer along with mRNA encoding N-Myc with or without mouse WT/S-A Ascl1, as indicated. (A) Embryos were subject to *in situ* hybridisation for AVNA markers as indicated at stage 18-19. (B) Embryos were scored for marker expression comparing the injected and uninjected side (see supplementary material Fig. S3 for scoring scheme). For scoring data, *n*=24-32. Kruskal–Wallis non-parametric ANOVA was performed comparing WT ASCL1 and S-A ASCL1 with and without N-Myc (*P<0.05, **P<0.0005 and ***P<0.000005).

With this in mind, we investigated whether overexpression of *MYCN* affected AVNA differentiation. N-Myc overexpression alone had little effect on expression of endogenous Hand2, Phox2a or TH (Fig. 7), indicating that increased expression of N-Myc cannot perturb the normal differentiation of AVNA cells. Next, in order to mimic the situation found in some NB cells, in which both ASCL1 and N-Myc protein are highly expressed, we co-injected N-Myc with levels of WT *Ascl1* mRNA that would induce ectopic AVNA cells. Despite having little effect on the formation of endogenous AVNA cells, N-Myc overexpression perturbed Ascl1-induced ectopic AVNA differentiation, significantly reducing ectopic Hand2 and TH expression (Fig. 7A), reducing overexpression of these markers from at least 75% of embryos to under 25% (Fig. 7B). Phox2a staining was relatively unchanged in the majority of the embryos analysed. Thus, in the presence of elevated levels of Ascl1, N-Myc suppresses AVNA cell differentiation as shown by a reduction in Hand2 and TH staining. To determine how the SP phospho-status of Ascl1 affected the ability of N-Myc to inhibit Ascl1-induced ectopic AVNA differentiation, S-A *Ascl1* mRNA was co-injected with *N-Myc* mRNA. In contrast to WT Ascl1, S-A Ascl1 can still induce AVNA cells in the presence of elevated N-Myc. Hence, high levels of N-Myc can inhibit differentiation of AVNA cells driven by elevated WT Ascl1 but not by elevated S-A Ascl1 (Fig. 7A,B). This indicates that the suppression of differentiation by N-Myc acts at least partially via the regulated phosphorylation of Ascl1.

## DISCUSSION

NB seems to be an undifferentiated tumour of the sympathetic nervous system based upon its histological phenotype and its ability to undergo further neuronal differentiation and maturation with retinoic acid treatment (Thiele et al., 1985). However, although NB cells do maintain proliferative potential, they cannot be simply viewed as very early sympathetic precursor cells because they also express many of the markers that are associated with differentiated NA neurons, including Phox2a, Hand2 and TH. Indeed, NA neurons of the sympathetic nervous system are unique during normal development in their ability to express markers of differentiated NA neurons while simultaneously retaining their proliferative potential (Ernsberger et al., 1989). Given this, NB might derive from cells that have begun to express markers of the sympathetic nervous system, but have failed to progress past this point to full maturity and more importantly have failed to exit the cell cycle. If this is the case, studying the developmental stage from which NB arises during development might yield vital insight into both NB pathogenesis and potential modes of treatment. However, the inaccessibility and migratory nature of these cells has made their characterisation particularly problematic in mammalian systems. In this study we have shown that the AVNA cells in *Xenopus*, which were first identified by Parlier and colleagues (2008), represent a useful model to investigate mechanisms contributing to NB formation and maintenance. Moreover, elucidation of crucial mechanisms regulating these cells provides a platform to explore new therapeutic approaches for this devastating disease.

We find that *Xenopus* AVNA cells share characteristics of NB cells. Firstly, these cells are derived from migrating neural crest and express key markers of sympathetic nervous system development. When migration of these neural crest precursors to the anteroventral region is prevented by dnSlug, the neural crest cells that remain at the neural folds do not express the markers of NA neurons. This failure to differentiate is potentially the result of improper extracellular signalling in the anteroventral region; for example, BMP2 signals, which are required for normal NA development in mammals (Howard, 2005) and for AVNA cells in *Xenopus* (Parlier et al., 2008) are not received. This is particularly interesting because BMP2 signalling has also been shown to play a role in differentiation of NB cells (Nakamura et al., 2003). Secondly, these AVNA cells express known NA markers also found in NB cells, such as Hand2, Phox2a and TH. AVNA and NB cells also express Ascl1. Ascl1 is only expressed transiently in AVNA differentiation (Parlier et al., 2008) (Fig. 2), whereas NB tumours and almost all NB cell lines have high levels of ASCL1 expression, indicating that NB cells are stalled at this transient stage of NA neuron development. This observation allows us to pinpoint much more closely the developmental window from which NB cells are likely to arise. Interestingly, ASCL1 levels are found to rapidly decrease when NB cells continue differentiation upon treatment with retinoic acid (Söderholm et al., 1999), supporting the hypothesis that NB cells are trapped in a transient phase of development. Thirdly, *Xenopus* primary neurons require cell cycle exit to undergo differentiation (Vernon et al., 2003), whereas differentiation of AVNA cells show a reduced requirement for this (Fig. 3). Nevertheless, increasing CDK levels does suppress differentiation of both endogenous AVNA cells and ectopic neurons generated after Ascl1 overexpression (Fig. 6).

Ascl1 is a powerful proneural transcription factor, driving both extensive neurogenesis in ectoderm and in neural precursors of the CNS (Ali et al., 2014; Castro et al., 2011), as well as playing a key role in a transcription factor cocktail that can reprogramme mammalian fibroblasts directly into neurons (Pang et al., 2011; Vierbuchen et al., 2010). Why would such a potent inducer of neuronal differentiation be elevated in NB where these cells fail to adopt their developmental fate and instead proliferate with little control? Recent evidence has shown that, at endogenous levels found at specific stages of development, Ascl1 supports and indeed is essential for proliferation of neural stem cells in the CNS (Castro et al., 2011). However, higher levels of Ascl1 result in cell cycle exit and differentiation (Ali et al., 2014; Castro et al., 2011). We see that the ability of Ascl1 to drive neuronal differentiation in development is not only controlled by the level of the protein but also by its phospho-status (Ali et al., 2014) (Figs 5-7), and that Ascl1 can be phosphorylated by CDKs in a cell-cycle-dependent manner (Ali et al., 2014).

Much of Ascl1's ability to induce expression of NA markers is probably due to an indirect transcriptional cascade. None of the NA markers (Phox2a, Hand2 or TH) has been shown to be a direct downstream target of Ascl1 (Castro et al., 2011), but they are upregulated sequentially during development (Howard, 2005; Rohrer, 2011). For instance, Hand2 is particularly important for the generation of NA neurons because it regulates expression of TH (Lei and Howard, 2011). Although the direct connections between Ascl1 and neuronal differentiation in the peripheral nervous system is less clear than in the CNS, Ascl1 overexpression does result in expression of NA markers in *Xenopus* embryos (Figs 6 and 7), suggesting some analogy between the function of Ascl1 in the peripheral and central nervous systems.

Ascl1 is found in multiply phosphorylated forms in almost all NB cell lines (Fig. 5). Hence, we sought to use the *Xenopus* AVNA system to investigate the effect of elevated CDK levels on AVNA cell differentiation and whether effects seen are mediated by phosphorylation of Ascl1 in the developing embryo. We see that elevated CDK suppresses differentiation of AVNA cells and this is dependent on Ascl1-dependent phosphorylation (Fig. 6). This is consistent with a model whereby high levels of CDK, characteristic of NB cells (Molenaar et al., 2008), not only promote cell cycling but also inhibit NA cell differentiation directly via phosphorylation of endogenous Ascl1. Sympathetic progenitor cells are known to maintain their proliferative potential while expressing NA markers such as Ascl1, Phox2a, Hand2 and TH (Ernsberger et al., 1989; Rohrer, 2011), although we observed decreased expression of these markers on CDK hyperactivation (Fig. 6). Hence, lower levels of CDK activity that will be required to support progenitor maintenance seem to be permissive for NA gene expression. However, higher CDK levels produced in our overexpression studies did suppress expression of these NA markers, and a similar situation is potentially found in NB cell tumours. Little is known about the effects of slowing (as opposed to arresting) the cell cycle on differentiation in the PNS, but, during development of CNS neurons, an initial slowing of the cell cycle rather than a complete halt ultimately promotes neuronal differentiation (Lange et al., 2009). This raises the possibility of using low levels of CDKIs to promote differentiation during NB treatment (Molenaar et al., 2008; Rader et al., 2013).

N-Myc has little effect on AVNA cell differentiation during normal development, when Ascl1 is only transiently expressed. However, ectopic N-Myc inhibits the ability of cells expressing elevated Ascl1 from undergoing AVNA differentiation and is perhaps analogous to the situation in NB, where both N-Myc and Ascl1 levels remain high. N-Myc-mediated inhibition of differentiation in AVNA cells also occurs via a mechanism involving Ascl1 phosphorylation on SP sites. This is consistent with N-Myc being found in the most aggressive NBs and its inverse association with differentiation *in vitro* (Thiele and Israel, 1988; Thiele et al., 1985).

Taken together, our data not only identify a developmentally relevant stage at which stalled differentiation might result in NB cell formation but also suggest a possible mechanism for suppression of NA differentiation within this population. NA cells with perhaps abnormally high or prolonged CDK activity, although passing through the developmental window of high ASCL1 expression, will result in the maintenance of ASCL1 protein in its highly phosphorylated form. This hyper-phosphorylated ASCL1 might play a direct role in promoting ‘stem-ness’ in the NA precursor population, and prevent these cells from undergoing further differentiation [a process that requires un(der)phosphorylated ASCL1]. If cells fail to pass through the developmental window of transient high ASCL1 expression, followed by differentiation and subsequent ASCL1 downregulation, these elevated levels of phosphorylated ASCL1 would cause NB precursors to remain in cycle, and cells would be able to accumulate further epigenetic or genetic ‘hits’ such as *MYCN* amplification and chromosomal rearrangements, driving tumour progression. Moreover, N-Myc amplification will further drive proliferation, potentially by feeding the maintenance of phospho-ASCL1.

The advancement of *Xenopus* as a model to gain a greater understanding of the cell cycle and transcriptional events of NA cell formation will surely provide a very versatile system, not only to study normal NA neuron differentiation, but it will also act as a uniquely tractable model to explore mechanisms that can perturb or promote this differentiation in disease and its therapy.

## MATERIALS AND METHODS

### *Xenopus laevis* extracts and embryos

Acquisition of *Xenopus laevis* eggs and embryos, preparation and injection of synthetic mRNA and DNA MOs, staging of embryos, and *in situ* hybridisation were undertaken as described previously (Vernon et al., 2003; Vosper et al., 2007, 2009). Scoring standards for each marker are shown in supplementary material Fig. S3. *Xenopus* experiments were carried out according to institutional standards of the University of Cambridge and under licence from the UK Government Home Office.

### Cell culture and western blotting

NB cells were cultured in RPMI-1640 (Gibco) with 10% fetal bovine serum (Atlanta Biologicals), 1% Glutamax (Gibco) and 100 units/ml penicillin/100 µg/ml streptomycin (Sigma). Cells were lysed with RIPA buffer [150 mM NaCl, 1.0% IGEPAL^®^ CA-630 (Sigma), 0.5% sodium deoxycholate, 0.1% SDS, and 50 mM Tris, pH 8.0] and treated with Lambda Protein Phosphatase (New England BioLabs) to determine ASCL1 phospho-shift. Following electrophoresis, membranes were incubated with an anti-ASCL1 [a kind gift from David Anderson and Francois Guillemot (Castro et al., 2011)] and anti-Gapdh (Sigma), and imaged using SuperSignal West Pico Chemiluminescent Substrate (Pierce).

### Quantitative real-time PCR (qPCR)

cDNA was generated from NB cells and 50 ng used per qPCR reaction in a Light-Cycler 480 PCR system with SYBR Green mix (Roche). *HPRT1* and *GAPDH* were used as house-keeping genes. Thermal cycling conditions: 95°C for 5 min, then 45 cycles of 95°C for 10 s, 60°C for 10 s and 72°C for 10 s. Primer sequences are in supplementary material Table S1.

### Generation of lentivirally transduced cell lines

Viral constructs were generated by site-directed mutagenesis using the QuikChange II XL Site-Directed Mutagenesis Kit (Agilent Technologies) and cloned into a 3rd Gen Lenti-X vector (Clontech) using In-Fusion^®^ HD Cloning Kit User Manual (Clontech). Viral constructs were made by transfecting HEK293 cells with 250 mM calcium phosphate and the vector of interest, and packaging mix: PMD2G, PMLg, REV/PRSV in a ratio of 6:3:4:2, respectively. Virus was titered using the Lenti-X™ qRT-PCR Titration Kit (Clontech). SY5Y NB cells were transduced with a Tet-On transactivator (LVX-Tet3G) at a multiplicity of infection (MOI) of 10. After 48 h, cells were selected with 500 µg/ml G418 for 72 h based on a previously determined optimal antibiotic kill curve. These cells were then transduced with LVX-TRE3G- encoding either human WT ASCL1 or S-A ASCL1 at an MOI of 10 and then selected after 48 h with 1 µg/ml puromycin for 72 h based on previously determined kill curves. Cells were induced with 1 µg/ml of doxycycline (Sigma).

### Microarray analysis of NB and normal tissues

Analysis was performed using R2: microarray analysis and visualisation platform (http://r2.amc.nl). Analysis was performed using the Megasampler algorithm to analyse expression across datasets. NB tumour data was from NB88 [R2 annotation: Tumor Neuroblastoma–Versteeg–88–MAS5.0–u133p2; accession no. GSE16476] and normal tissue data was from Roth504 [Normal Various–Roth–504–MAS5.0–u133p2; accession no. GSE7307]. Expression levels were normalised using the MASS5.0 algorithm (Affymetrix, Inc.).

## Supplementary Material

Supplementary Material
