# Supplementary material for: Ascl1 phospho-status regulates neuronal differentiation in a Xenopus developmental model of neuroblastoma
Source: Dis Model Mech. 2015 May 1;8(5):429–41. doi: 10.1242/dmm.018630 (PMC4415893; doi:10.1242/dmm.018630)
Supplement: Supplementary Material [file supp_8_5_429__index.html]

Ascl1 phospho-status regulates neuronal differentiation in a Xenopus developmental model of neuroblastoma — Supplementary Material 

# Ascl1 phospho-status regulates neuronal differentiation in a *Xenopus* developmental model of neuroblastoma

## DMM018630 Supplementary Material

**Files in this Data Supplement:**

- **Supplementary Material**
