# Supplementary material for: Ascl1 phospho-status regulates neuronal differentiation in a Xenopus developmental model of neuroblastoma
Source: Dis Model Mech. 2015 May 1;8(5):429–41. doi: 10.1242/dmm.018630 (PMC4415893; doi:10.1242/dmm.018630)
Supplement: Supplementary Material [file supp_8.5.429_DMM018630.pdf]

Figure. S1. Sox10 expression on the dorsal region of Xenopus embryos

Xenopus embryos were fertilised and allowed to develop to the indicated developmental stage before fixing and staining by in situ hybridisation for neural crest marker Sox10. Embryos are orientated with the dorsal side imaged and head to the top.

Figure. S2. Comparison of Ascl1 serine-proline sites across species

Ascl1 protein is illustrated schematically across species with serine-proline pairs indicated in green. Ascl1 phosphomutant forms were generated by performing site-directed mutagenesis changing all indicated serine-proline pairs to alanine-proline.

Figure. S3. Scoring standards for AVNA marker expression comparing the injected and uninjected sides

In situ hybridisation scoring standards for each marker are shown above. Each embryo from Figures 1, 3, 6 & 7 were assigned a numeric score based as indicated above.

Table S1. Primers used in qPCR analysis of gene expression.

Primers were designed for qPCR of Ascl1 in neuroblastoma cell lines. Primers were designed to have a Tm between 57ºC and 60ºC. HPRT1 and GAPDH were used as 'house-keeping' controls.

S1

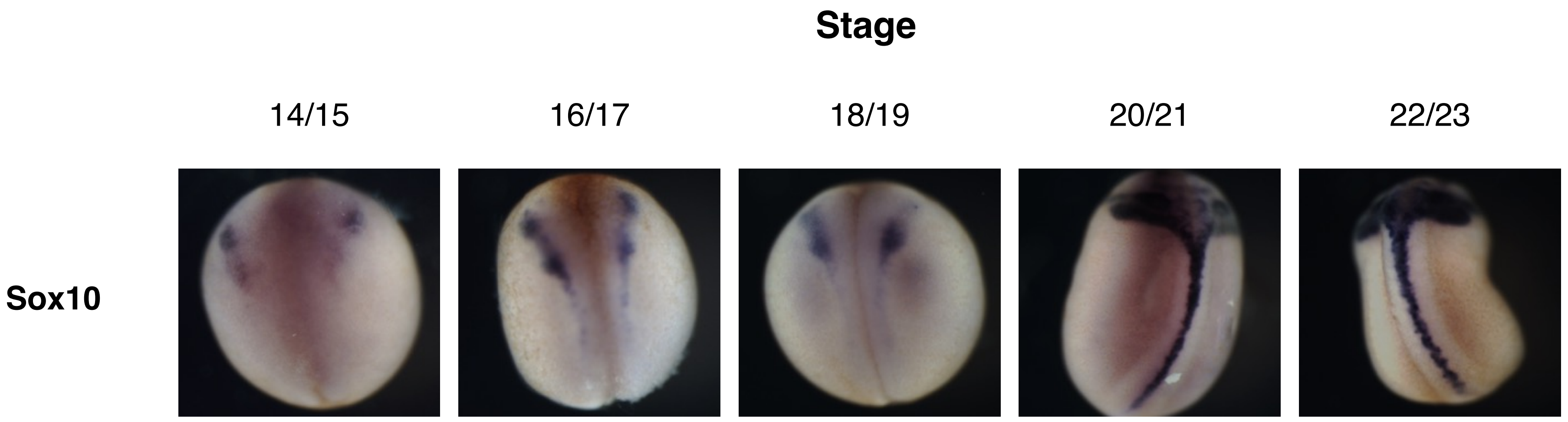

Figure S1

S2

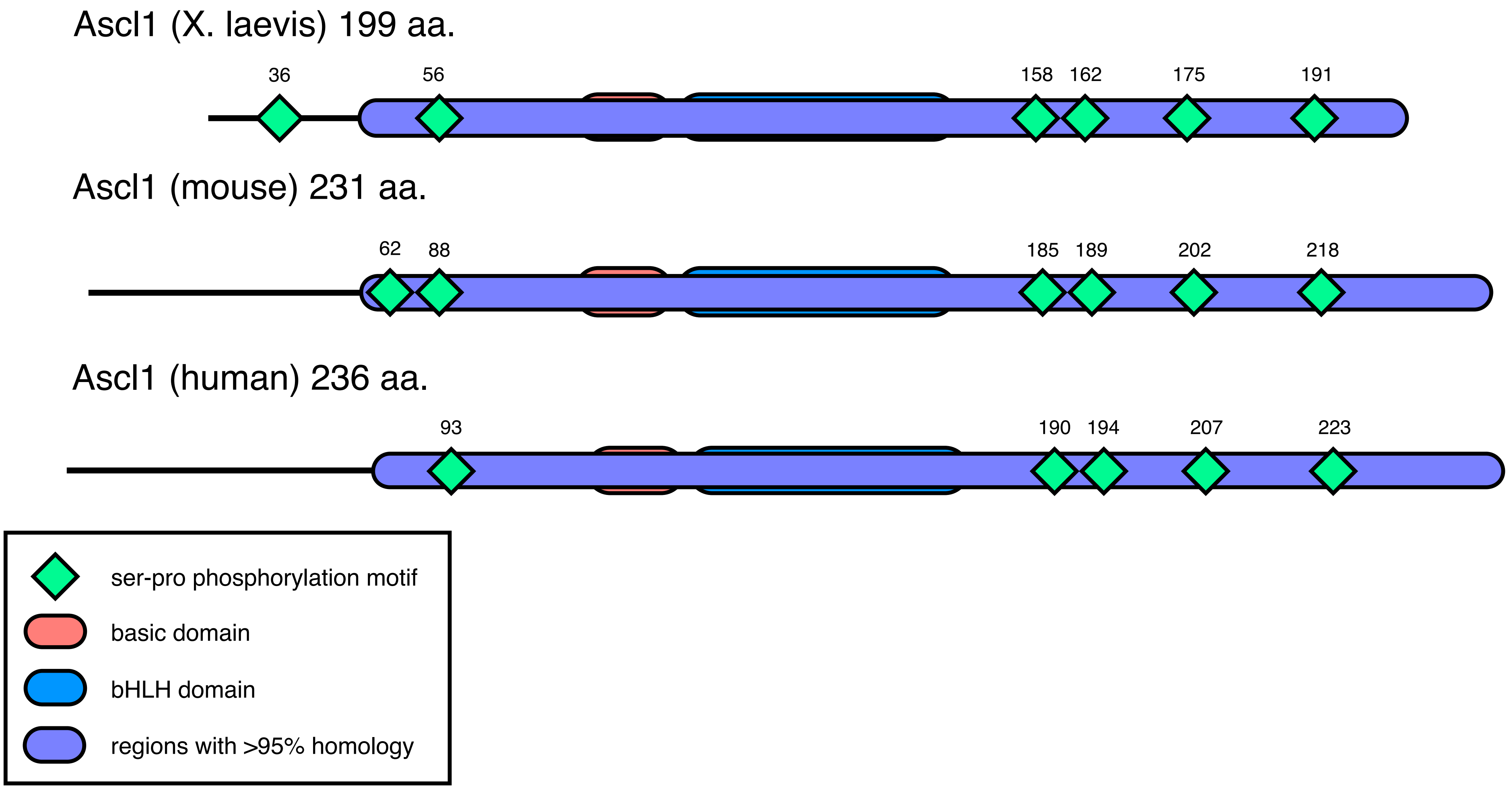

Figure S2

S3

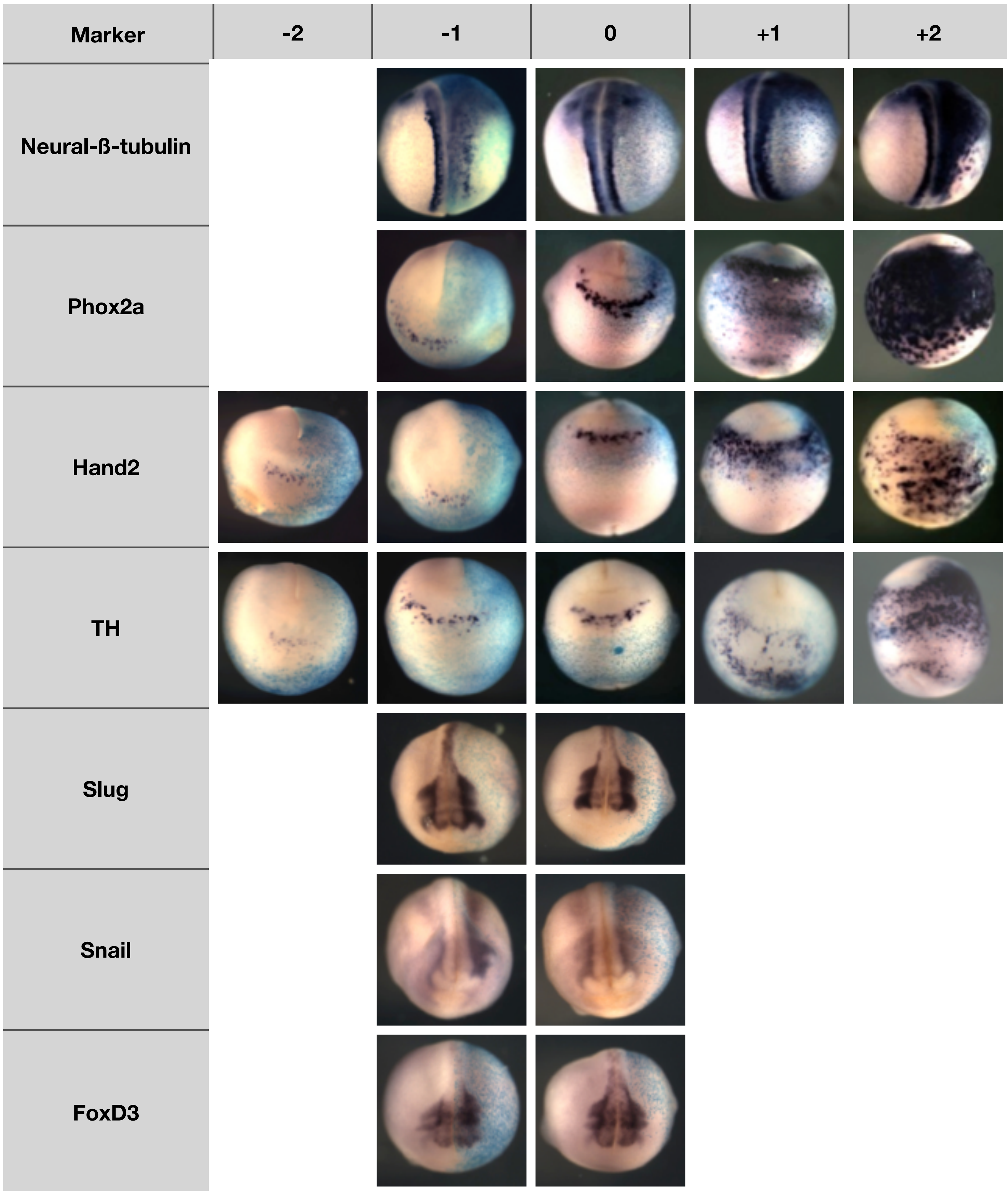
Marker
-2
-1
0
+1
+2
Neural-ß-tubulin
Phox2a
Hand2
TH
Slug
Snail
FoxD3

Figure S3

Gene	Forward/Reverse	Sequence
hASCL1	Forward	CATCTCCCCCAACTACTCCA
hASCL1	Reverse	AACGCCACTGACAAGAAAGC
HPRT1	Forward	TGGCGTCGTGATTAGTGATG
HPRT1	Reverse	ATCCAGCAGGTCAGCAAAG
GAPDH	Forward	GAAGGTGAAGGTCGGAGTC
GAPDH	Reverse	TGGAAGATGGTGATGGGATT

Table S1
